# Phase-amplitude coupling between infraslow and high-frequency activities well discriminates between the preictal and interictal states

**DOI:** 10.1038/s41598-021-96479-1

**Published:** 2021-08-31

**Authors:** Hiroaki Hashimoto, Hui Ming Khoo, Takufumi Yanagisawa, Naoki Tani, Satoru Oshino, Haruhiko Kishima, Masayuki Hirata

**Affiliations:** 1 Department of Neurological Diagnosis and Restoration, Graduate School of Medicine, Osaka University, Yamadaoka 2-2, Suita, Osaka 565-0871 Japan; 2 Department of Neurosurgery, Otemae Hospital, Osaka, Osaka 540-0008 Japan; 3 Department of Neurosurgery, Graduate School of Medicine, Osaka University, Suita, Osaka 565-0871 Japan

**Keywords:** Epilepsy, Epilepsy

## Abstract

Infraslow activity (ISA) and high-frequency activity (HFA) are key biomarkers for studying epileptic seizures. We aimed to elucidate the relationship between ISA and HFA around seizure onset. We enrolled seven patients with drug-resistant focal epilepsy who underwent intracranial electrode placement. We comparatively analyzed the ISA, HFA, and ISA-HFA phase-amplitude coupling (PAC) in the seizure onset zone (SOZ) or non-SOZ (nSOZ) in the interictal, preictal, and ictal states. We recorded 15 seizures. HFA and ISA were larger in the ictal states than in the interictal or preictal state. During seizures, the HFA and ISA of the SOZ were larger and occurred earlier than those of nSOZ. In the preictal state, the ISA-HFA PAC of the SOZ was larger than that of the interictal state, and it began increasing at approximately 87 s before the seizure onset. The receiver-operating characteristic curve revealed that the ISA-HFA PAC of the SOZ showed the highest discrimination performance in the preictal and interictal states, with an area under the curve of 0.926. This study demonstrated the novel insight that ISA-HFA PAC increases before the onset of seizures. Our findings indicate that ISA-HFA PAC could be a useful biomarker for discriminating between the preictal and interictal states.

## Introduction

Epilepsy is a common neurological disorder. Timely detection of seizures is important for physicians to diagnose and quantitatively measure epilepsy. Infraslow activity (ISA) and high-frequency activity (HFA) are key biomarkers of seizure detection^1–6^. They are both included in wideband electroencephalogram measurements and are measured by intracranial electroencephalogram (iEEG). ISA, which is also referred to as a direct current (DC) shift^7^, is a low-frequency component. Modern alternating current amplifiers with a time constant of 10 s allow filter-setting to clinically record ISA (i.e., the cutoff frequency of a high-pass filter is above 0.016 Hz)^4^. HFA can be physiological^8,9^ or pathological^3,10^. Epileptic HFA is usually > 80 Hz^3^, and it is clinically important to distinguish between physiological and pathological HFAs in epileptic patients^11–13^. High-frequency oscillations (HFOs), which are a subgroup of HFA^6^, are isolated oscillations that stand out from the background and can be further classified as ripples (80–250 Hz) and fast ripples (250–500 Hz)^3,10,14^.

The relationship between HFA and ISA has not yet been established. Ictal HFA and ISA are likely to occur in the same contacts^1,4^. Both occur earlier than conventional iEEG changes, but ictal-ISA is observed more often and may occur earlier than HFA^2,4,15^. Ictal ISAs that precede ictal HFAs are called “active DC shifts,” whereas ictal ISAs that occur immediately after the onset of conventional ictal electroencephalogram (EEG) patterns and HFAs are called “passive DC shifts”^4^. In interictal states, ISA accompanied by HFA could be a useful surrogate marker of the epileptogenic zone––this is called a “red slow”^4,16^.

Phase-amplitude coupling (PAC) is used to investigate the relationship between the low- and high-frequency bands on the EEG^17^. PAC analysis measures the degree of synchronization between the phases with low-frequency oscillation and high-frequency amplitude^18^. Ictal HFA amplitudes coupled with δ^19,20^, θ^21,22^, and α^21^ phases, and β-HFA coupling are reported as useful markers for seizure detection^23^. Moreover, coupling θ waves with HFOs has been reported to well discriminate normal brain regions from the seizure onset zone (SOZ)^24^.

These pathological PACs are usually accompanied by ictal HFA, whereas physiological PACs that are involved in motor-related HFAs (i.e., high γ band) appear before HFA increases^25,26^. Physiological PAC precedes physiological HFA; however, whether a preceding seizure-related PAC can be observed before ictal HFA appears is unknown. Moreover, the coupling between a frequency of 0.1 Hz and HFA has been previously reported^19^, but whether a similar coupling between 0.016 Hz and HFA exists is unclear.

In our previous case report, we demonstrated that the PAC between ISA and HFA preceded seizure onset (SO)^27^. In this study, we hypothesized that ISA-HFA PAC may precede the SO and would aid clinicians in discriminating between the interictal and preictal states. The synchronization index (SI)^18^ was used to measure the strength of PAC between the HFA amplitude and the ISA phase.

## Results

Overall, we recorded 15 seizures, all of which were focal. We observed focal to bilateral tonic–clonic seizure in 12 (80%) episodes. Eleven (73%) seizures were observed during sleep. The number of SOZ contacts in each seizure is shown in Table 1. The contacts that showed initial epileptic changes immediately after the SO were determined as the SOZ contacts.

**Table 1:** Clinical profile of the enrolled patients.

Patient number	Sex	Age at surgery	Laterality	Pathology	Seizure number	Seizure type	Number of SOZ contacts	Wakefulness or sleep	Number of total implanted contacts
P1	Male	28	Right	MTLE	S1	FIAS, FBTCS	1	Wakefulness	96
					S2	FIAS, FBTCS	1	Sleep	
					S3	FIAS, FBTCS	1	Sleep	
					S4	FIAS, FBTCS	1	Wakefulness	
					S5	FIAS, FBTCS	1	Sleep	
P2	Male	47	Right	PLE	S1	FIAS, FBTCS	5	Sleep	54
P3	Male	20	Left	OLE^a^	S1	FIAS, FBTCS	2	Wakefulness	92
P4	Female	15	Left	MTLE	S1	FIAS, FBTCS	6	Sleep	72
					S2	FIAS, FBTCS	6	Sleep	
P5	Male	15	Right	OLE	S1	FIAS, FBTCS	4	Sleep	62
					S2	FIAS, FBTCS	4	Sleep	
					S3	FIAS, FBTCS	4	Sleep	
P6	Male	37	Left	MTLE	S1	FAS	3	Sleep	124
P7	Male	20	Right	MTLE	S1	FAS	2	Sleep	77
					S2	FAS	2	Wakefulness	

The seizure number is the serial number of seizures in each participant.

SOZ, seizure onset zone; MTLE, mesial temporal lobe epilepsy; FIAS, focal-impaired awareness seizure; FBTCS, focal-to-bilateral tonic–clonic seizure; PLE, parietal lobe epilepsy; OLE, occipital lobe epilepsy; FAS, focal aware seizure.

^a^Based on the presurgical examination, we suspected MTLE or OLE. The electrodes were primarily placed on the temporal lobe. Therefore, few electrodes were placed on the occipital lobe. The intracranial EEG study revealed OLE, and the range of locations where the intracranial electrodes were placed was not sufficiently wide to detect the exact seizure onset zone. As a result, we decided not to perform focal resection surgery.

**Figure 1:**
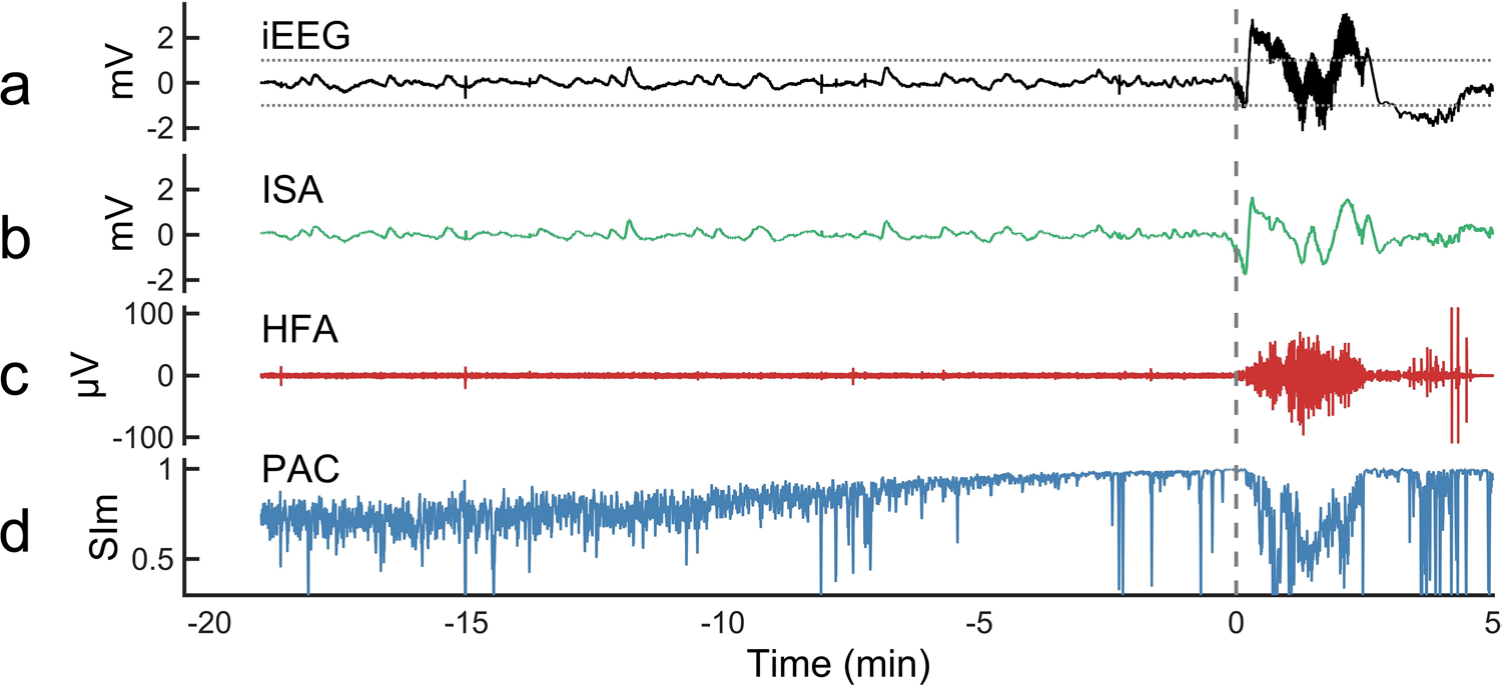
A representative seizure in a patient. Representative multimodal signals related to seizure (seizure 1 [S1] in Patient 1 [P1] shown in Table 1) were calculated from one contact placed in the SOZ. The data from 20 min before to 5 min after the SO (0 min) are shown. The top to bottom graphs display the raw iEEG signals in black (**a**), 0.016–1 Hz band-pass filtered signal as the ISA in green (**b**), 80–250 Hz band-pass filtered signals as the HFA in red (**c**), and ISA-HFA SIm as the PAC in blue (**d**). In the iEEG signals, ± 1 mV are indicated as horizontal dotted lines, which are the threshold for significant ISA related to seizures. After SO, activities over ± 1 mV exist in the iEEG signals, which are seizure-related ISA (**a**), and the same shape of signals is confirmed in ISA (**b**). Therefore, we knew that the 0.016–1 Hz band-pass filter enabled us to extract the ISA activity. At the same time, the HFA increased (**c**), which was seizure-related HFA. Before the SO, no activity for ISA or HFA occurred. However, the SIm started to increase from approximately 10 min before the SO and reached a peak at SO (**d**). For all figures: AUC, area under the curve; FWE, family-wise error; HFA, high-frequency activity; iEEG, intracranial electroencephalogram; ISA, infraslow activity; MRI, magnetic resonance imaging; nSOZ, non-seizure-onset zone; PCA, phase-amplitude coupling; SIm, magnitude of the synchronization index; SIp, phase of synchronization; SO, seizure onset; SOZ, seizure-onset zone.

**Figure 2:**
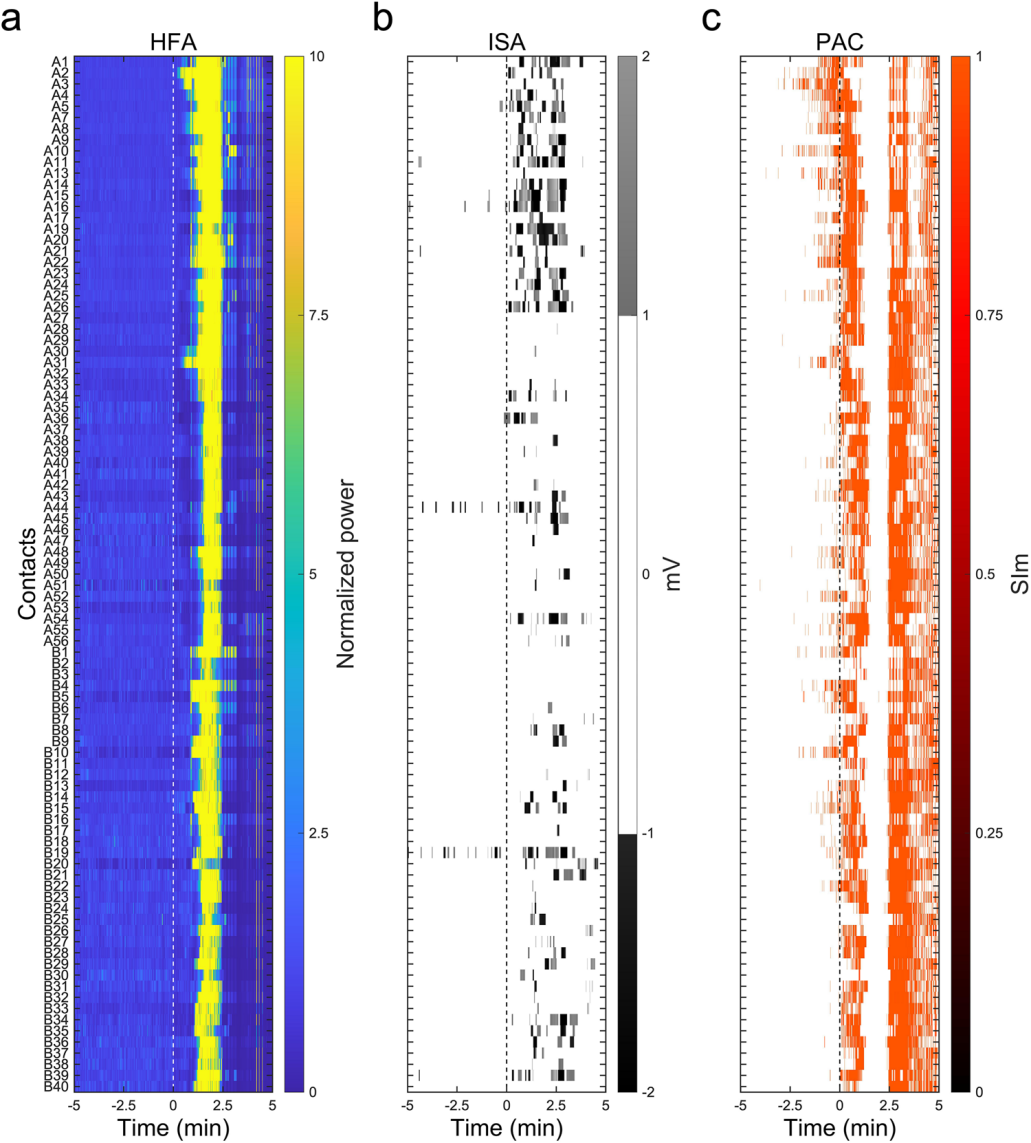
The topographies of HFA, ISA, and ISA-HFA SIm (PAC) of seizure 1 in Patient 1. The seizure-related data from 5 min before to 5 min after the SO (0 min) of seizure 1 (S1) in Patient 1 (P1) are shown. All contacts of P1 are shown. In Fig. 1, we only used the A2 contact that was placed in the SOZ. (**a**) The HFA-normalized power began increasing from the A2 contacts after SO and spread to other contacts. (**b**) Significant seizure-related ISAs, which are defined as more than 1 mV or less than − 1 mV, are indicated in grayscale. HFA and ISA occur after SO; however, no clear HFA or ISA occurs before SO. (**c**) Only significant SIm between the ISA phase and the HFA amplitude, which was obtained using the FWE-corrected threshold that was applied to the raw SIm, are shown. We observed significant ISA-HFA SIm, even before SO.

In Figs. 1 and 2, representative seizures of seizure 1 (S1) in Patient 1 (P1) indicated that ISA and HFA changes occurred after the SO, whereas no clear changes occurred in them before the SO. However, the magnitude of SI (SIm) started to increase, even a few minutes before the SO. The seizures in P1 have been reported in our previous case report^27^.

We were concerned that the SIm before SO may produce artificially high values; therefore, we evaluated the simulation signal (Supplementary Fig. S1a). The results revealed that SIm achieved high values intermittently more than 10 min before simulation of the HFA and ISA signals. This finding suggested that the SIm values were artificially high. However, when the same calculation was applied to the 60-min iEEG signal with no seizure, no high values occurred for ISA-HFA SIm (Supplementary Fig. S2b). Therefore, we concluded that the calculation of ISA-HFA SIm by using actual iEEG signals did not induce artificially high values.

In this study, we used a 1-s time window for calculating the SI. One period of the ISA frequency band was 62.5 s (i.e., 1/0.016); therefore, we were concerned that a short time window may induce artificially high values for ISA-HFA SIm. Then, we calculated the dynamic changes of ISA-HFA SIm around the SO using a 1-s, 30-s, and 63-s time window. The results from all seizures are displayed in Supplementary Fig. S3. We could observe that the longer the time window used, the lower ISA-HFA SIm values became (Supplementary Fig. S3 left panels, S4a). For comparison between SIm calculated using different time windows, ISA-HFA SIm values were normalized using averages and standard deviations calculated from the 60-min iEEG data that contained each seizure. The tendency that ISA-HFA SIm started to increase before SO was observed in all time window groups, and the shapes of plots were similar among groups (Supplementary Fig. S3 right panels). Normalized ISA-HFA SIm at SO showed no significant differences between the 1-s, 30-s, and 63-s time window (Supplementary Fig. S4b). We surmised that the results showing the same tendency regardless of the length of time window could be obtained because we applied a finite-impulse response (FIR) filter to the 60-min iEEG data before extracting the phase data and then cutting the ISA phase data into 1-s data. However, we suspected that the procedure would not be valid for cutting the iEEG data into 1-s data, and then applying an FIR filter to the 1-s iEEG data. In this study, we judged that a 1-s time window could be available for calculation of ISA-HFA SIm. Because we were concerned about worsening time resolution owing to averaging effect using a long time window, we decided to use the 1-s time window for ISA-HFA SI.

In addition, we found that, if the iEEG data were contaminated by external high-frequency noise, ISA-HFA SIm achieved artificially high values (Supplementary Fig. S1b). Therefore, at the preprocessing stage, we excluded from further analyses the iEEG data files that included severe external high-frequency noise. In this study, we defined severe external noise as high-frequency and high-amplitude activities that ranged over many contacts and stood out from background activities. We visually detected and manually removed these.

### Profiles of HFA, ISA, and PAC

**Figure 3:**
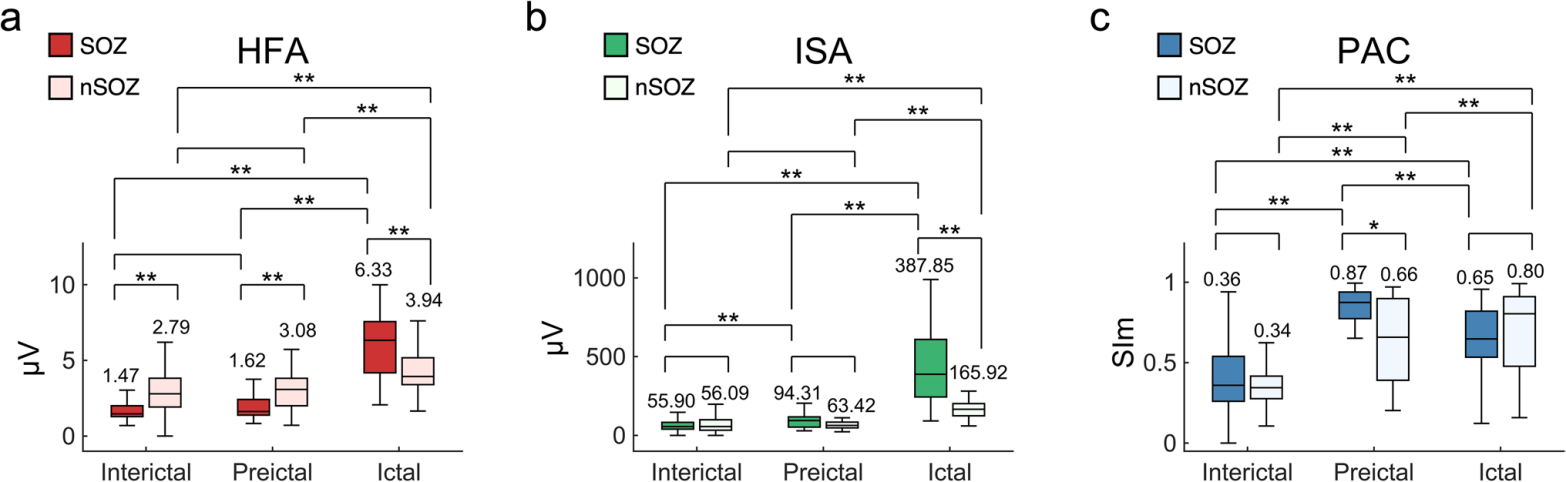
The profiles of HFA, ISA, and PAC. Results calculated from contacts placed in the SOZ and nSOZ are shown as box-and-whisker plots in which the median values at each group are displayed. (**a**) HFA amplitude of ictal-SOZ contacts achieved its maximum value and was significantly higher than that of interictal-SOZ contacts, preictal-SOZ contacts, and ictal-nSOZ contacts. (**b**) ISA oscillations of ictal-SOZ contacts achieved their maximum values and are significantly higher than those of interictal-SOZ contacts, preictal-SOZ contacts, and ictal-nSOZ contacts. (**c**) For PAC (i.e., ISA-HFA SIm), the SIm of the preictal-SOZ contacts achieves its maximum value and is significantly higher than that of interictal-SOZ contacts and ictal-SOZ contacts. Median values of each group are indicated as numerical values and the center mark on each box. *Corrected p < 0.05, ** corrected p < 0.01, Wilcoxon rank-sum test or Wilcoxon signed-rank test across contacts, nine multiple comparison at each group corrected by Bonferroni method.

We averaged and compared the HFA amplitude, ISA oscillation, and PAC (i.e., ISA-HFA SIm) across the SOZ and non-SOZ (nSOZ) contacts within the three states (Fig. 3). In ictal-HFA and ictal-ISA, SOZ contacts showed significantly higher values than did the nSOZ contacts (corrected *p* = 1.53 × 10^–4^ in HFA, 6.80 × 10^–8^ in ISA; two-tailed Wilcoxon rank-sum test with Bonferroni correction) (Fig. 3a,b); however, there were no significant differences in ictal-PAC between the SOZ contacts and the nSOZ contacts (Fig. 3c). The ictal-HFA and ictal-ISA of the SOZ contacts achieved their maximum value, which was significantly higher than the interictal-HFA and preictal-HFA, and interictal-ISA and preictal-ISA of the SOZ contacts (corrected *p* = 1.67 × 10^–23^ in HFA, 1.19 × 10^–24^ in ISA; two-tailed Wilcoxon rank-sum test between interictal and ictal stages, and corrected *p* = 1.01 × 10^–7^ in HFA, 1.01 × 10^–7^ in ISA; two-tailed Wilcoxon signed-rank test between the preictal and ictal states with Bonferroni correction) (Fig. 3a,b). However, the preictal-PAC of the SOZ contacts achieved its maximum value and was significantly higher than the interictal- and ictal-PAC of the SOZ contacts (corrected *p* = 2.81 × 10^–19^; two-tailed Wilcoxon rank-sum test between interictal and preictal states, and corrected *p* = 9.06 × 10^–6^; two-tailed Wilcoxon signed-rank test between preictal and ictal states with Bonferroni correction) (Fig. 3c).

Contrary to our expectation, the interictal-HFA and preictal-HFA of the SOZ contacts were significantly lower than those of nSOZ contacts (corrected *p* = 7.89 × 10^–38^ in interictal, 1.72 × 10^–3^ in preictal; two-tailed Wilcoxon rank-sum test with Bonferroni correction).

### The timepoint when significant changes occurred

**Figure 4:**
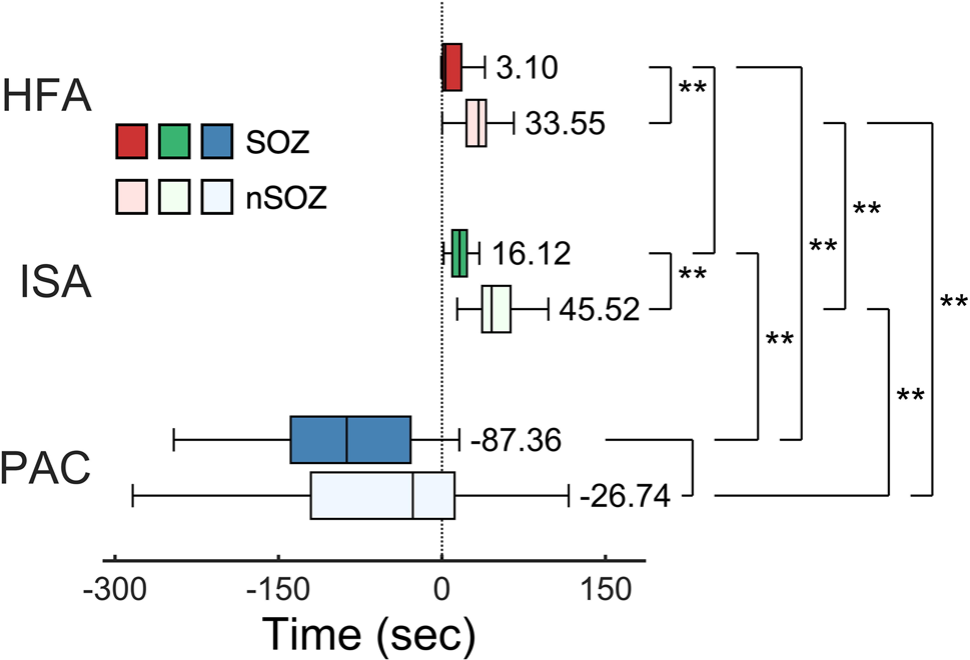
The timepoints at which significant changes occurred. Timepoints at which significant seizure-related changes occur are shown in the box-and-whisker plots. Median timepoints of each group are indicated as numerical values and the center mark on each box. For HFA and ISA, the change in the SOZ contacts occurs significantly before that of the nSOZ contacts. The time of PAC (i.e., ISA-HFA SIm) is earlier than that of SO (0 s). No significant differences exist between the times of SOZ-PAC and nSOZ-PAC. **Corrected p < 0.01, Wilcoxon rank-sum test across contacts, nine multiple comparison corrected by Bonferroni method.

Across the SOZ and nSOZ contacts, we compared the timepoints when HFA, ISA, and PAC (i.e., ISA-HFA SIm) showed significant changes related to seizures (Fig. 4). The HFA and ISA occurred significantly earlier in the SOZ contacts than in the nSOZ contacts (corrected *p* = 2.17 × 10^–6^ in HFA, 8.13 × 10^–10^ in ISA; two-tailed Wilcoxon rank-sum test with Bonferroni correction), whereas for PAC no significant differences existed between the SOZ contacts and nSOZ contacts. There were no significant differences between SOZ-HFA and SOZ-ISA. However, the SOZ-PAC changed at a significantly earlier time (median, − 87.36 s) than did the SOZ-HFA (median, 3.10 s) and the SOZ-ISA (median, 16.12 s) (corrected *p* = 5.27 × 10^–12^ in HFA, 3.65 × 10^–12^ in ISA; Wilcoxon rank-sum test with Bonferroni correction).

### The percentages at which significant changes occurred

We evaluated the percentages of the appearance of significant HFA, ISA, and PAC (i.e., ISA-HFA SIm) across patients. Significant changes in HFA and PAC occurred in nearly all SOZ and nSOZ contacts. For ISA, 89% of SOZ and 67% of nSOZ contacts showed significant changes. There were no significant differences between groups (Wilcoxon signed-rank test with Bonferroni correction) (Supplementary Fig. S5).

### Phase-based analysis

**Figure 5:**
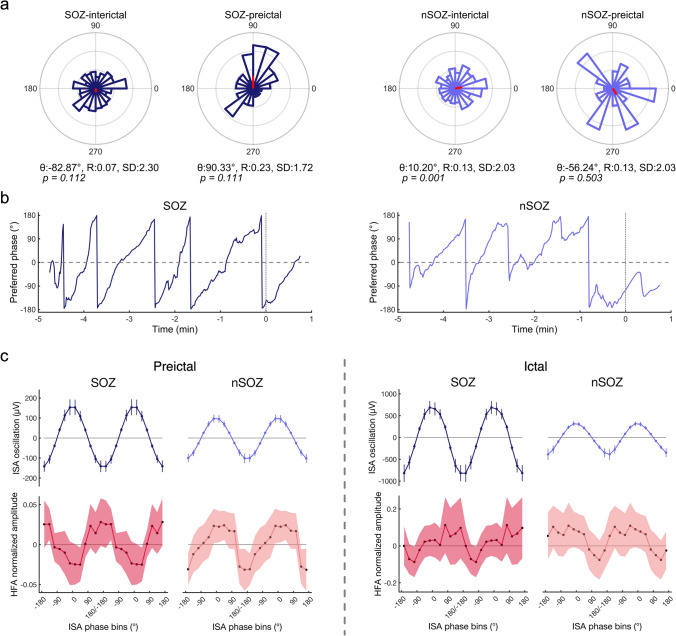
Phase-based analyses of PAC. (**a**) We calculated the average values of the SIp of SOZ contacts or nSOZ contacts in the interictal and preictal states. No similarities existed between the SOZ contacts and nSOZ contacts. The angle (θ), length (R), and standard deviation (SD) of the mean vector are indicated, and p values, calculated using the Rayleigh test, are shown. (**b**) We sequentially plotted the angles of the mean vector from − 5 to 1 min. We observed continuous and periodic shapes in SOZ contacts and discontinuous shapes in nSOZ contacts. (**c**) The SOZ contacts showed that the phase-tuning HFA-normalized amplitude is at its peak at the trough of the ISA oscillations and at its lowest during the peak of the ISA oscillations in the preictal states; this trend was reversed in nSOZ contacts. In the ictal states, these patterns became obscure. The error bars indicate 95% confidence intervals.

We used phase-based analyses to investigate the differences between SOZ-PAC and nSOZ-PAC (i.e., ISA-HFA SIm). Using a 30-s duration in the interictal or preictal states, we calculated the mean vectors of preferred phase of synchronization (SIp) in the SOZ contacts or the nSOZ contacts (Fig. 5a); there were no similarities between the SOZ and nSOZ in these two states. We observed significant nonuniformity only in the nSOZ-interictal state (*p* = 0.001, Rayleigh test).

We sequentially plotted the angle (°) of mean vectors calculated from SIp in SOZ contacts or nSOZ contacts from − 5 to + 1 min around the SO (Fig. 5b). In the SOZ contacts, we documented continuous and periodic angle changes from − 180° to + 180°, while angle changes in nSOZ contacts showed discontinuous and collapsed periodicity.

Figure 5c depicts ISA oscillations and HFA-normalized amplitude tuned by the phases of ISA, in which “preictal” indicates the average of 5 min (i.e., − 5 min to 0 min) and “ictal” indicates the average of 1 min (i.e., 0 min to 1 min). During the preictal states in the SOZ contacts, the HFA amplitude peaked at the trough of the ISA oscillation, whereas we found the opposite trend in the nSOZ contacts in that the HFA amplitude peaked at the peak of the ISA oscillation. These clear patterns were disrupted in the ictal state.

### Correlation between HFA and PAC

HFA increased in the ictal states, whereas PAC increased and displayed clear patterns in the preictal state, compared to the ictal state. After observing these contrasting results, we investigated the correlation between HFA and PAC.

By using all implanted contacts, we calculated the correlation coefficients (*r*) and their corrected p values with Bonferroni correction in combination with sequential HFA-normalized amplitude and sequential PAC (i.e., ISA-HFA SIm) from − 5 to + 2 min around the SO and displayed them as a matrix (Supplementary Fig. S6). We observed a positive correlation between the ictal-HFA (i.e., after 0 min) and the preictal-PAC (i.e., before 0 min). This finding demonstrated that contacts that indicated increased PAC before SO showed more of an increase in HFA after SO. Moreover, we observed a negative correlation along the diagonal line from approximately 0 min to 1.5 min, which corresponded to a correlation between ictal-HFA and ictal-PAC (red dashed square in Supplementary Fig. S6a).

### Classification

**Figure 6:**
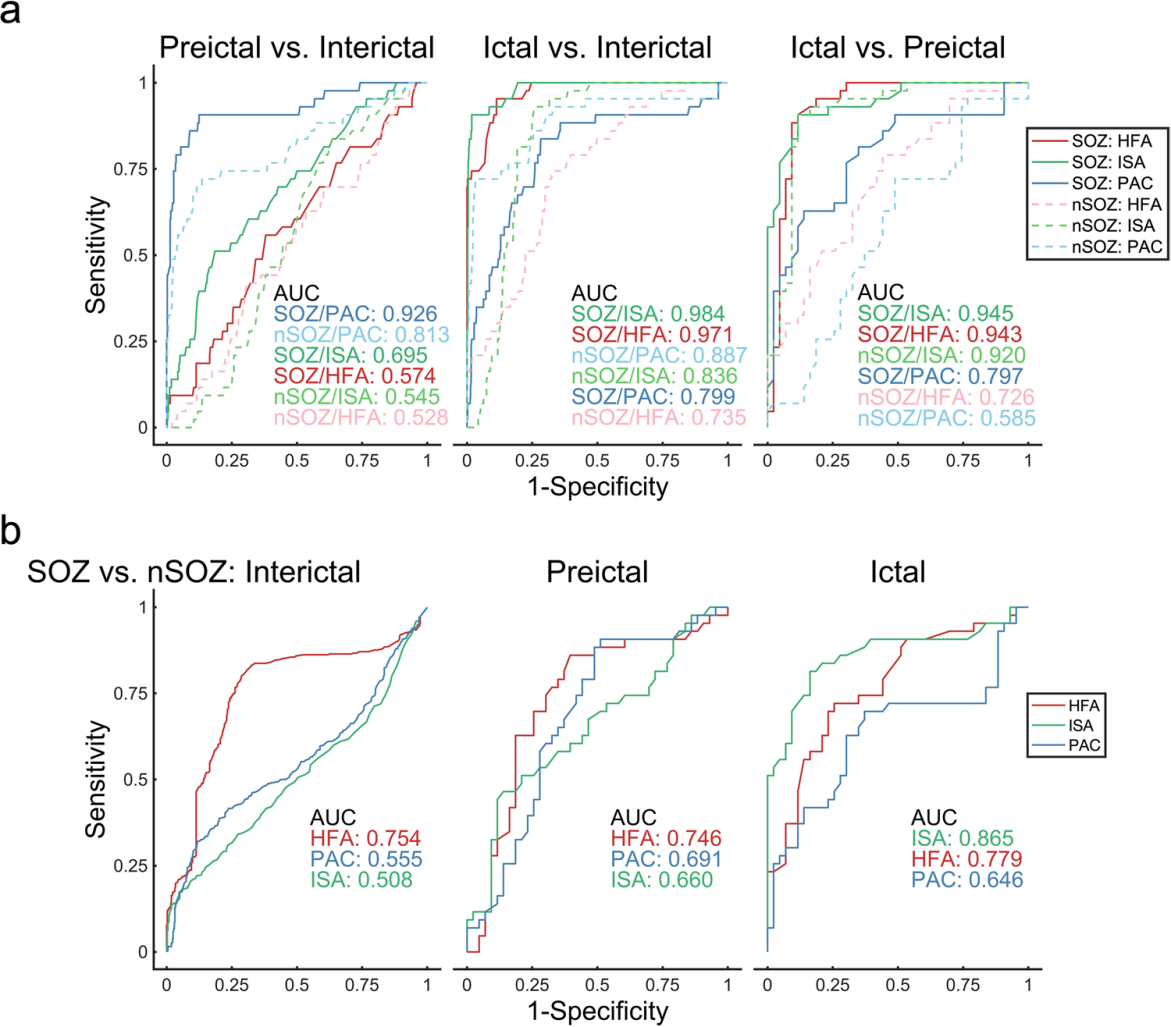
Average receiver operating characteristic curves of the results, after stage classification. (**a**) Preictal vs. interictal, ictal vs. interictal, and ictal vs. preictal stages are classified using HFA amplitude, ISA, and PAC (i.e., ISA-HFA SIm) of the SOZ contacts or the nSOZ contacts. SOZ-PAC shows the highest performance between the preictal and interictal states, whereas SOZ-ISA has a higher potential to distinguish between the ictal and interictal states and between the ictal and preictal states. (**b**) On classifying SOZ and nSOZ using HFA, ISA, and PAC, we found that HFA has the maximum AUC in the interictal and preictal states. ISA performs best in the ictal states.

To evaluate whether the HFA amplitude, ISA, and PAC (i.e., ISA-HFA SIm) accurately discriminates between the interictal, preictal, and ictal states, we compared the preictal state with the interictal state, the ictal state with the interictal state, and the ictal state with the preictal state by using SOZ contacts or nSOZ contacts (Fig. 6a). After classifying the preictal and interictal states, we found that the area under the curve (AUC) of SOZ-PAC was at its maximum and significantly higher than those of SOZ-ISA, SOZ-HFA, and nSOZ-PAC (corrected *p* = 1.69 × 10^–144^ in SOZ-ISA, 3.51 × 10^–157^ in SOZ-HFA, 4.85 × 10^–152^ in nSOZ-PAC; two-tailed Wilcoxon signed-rank test, three multiple comparisons corrected by Bonferroni method). SOZ-ISA showed the best performance in the comparisons of the ictal states with the interictal state and the ictal state with the preictal state; its performance was better than that of SOZ-HFA, SOZ-PAC, and nSOZ-ISA (between ictal and interictal, corrected *p* = 8.24 × 10^–25^ in SOZ-HFA, 1.02 × 10^–160^ in SOZ-PAC, 7.87 × 10^–78^ in nSOZ-ISA; between ictal and preictal, corrected *p* = 7.57 × 10^–12^ in SOZ-HFA, 4.23 × 10^–153^ in SOZ-PAC, 0.46 in nSOZ-ISA; two-tailed Wilcoxon signed-rank test, three multiple comparisons of each group corrected by Bonferroni method).

Finally, we classified SOZ and nSOZ using HFA, ISA, and PAC (i.e., ISA-HFA SIm) (Fig. 6b). In the interictal states, PAC and ISA showed a chance level; however, HFA performed better than the others (corrected *p* = 7.00 × 10^–159^ in HFA-ISA, 3.05 × 10^–154^ in HFA-PAC; two-tailed Wilcoxon signed-rank test, two multiple comparisons corrected by Bonferroni method). The HFA similarly showed the best performance in the preictal states (corrected *p* = 5.27 × 10^–83^ in HFA-ISA, 7.04 × 10^–45^ in HFA-PAC; two-tailed Wilcoxon signed-rank test, two multiple comparisons corrected by Bonferroni method). However, in the ictal states, the ISA showed the best performance (corrected *p* = 4.16 × 10^–84^ in ISA-HFA, 5.66 × 10^–155^ in ISA-PAC; two-tailed Wilcoxon signed-rank test, two multiple comparisons corrected by Bonferroni method).

## Discussion

In this study, we hypothesized that ISA-HFA PAC would precede the SO and could help clinicians discriminate between the interictal and preictal states. By extracting ISA using a 0.016–1 Hz bandpass filter and HFA by using an 80–250 Hz bandpass filter, we demonstrated that, in the ictal state, the SOZ contacts achieved significantly higher values in the HFA amplitude and the ISA oscillations than did the nSOZ contacts. HFA and ISA are often observed during seizures^15,28–30^ and they are useful biomarkers to detect the SOZ^4,31,32^. We also showed that, in the ictal state, the SOZ contacts achieved significantly higher values in the HFA amplitude and the ISA oscillations than did the nSOZ contacts. The onset of ictal-ISA is typically earlier than that of the HFA^15,32^; however, we did not observe any differences between the onsets of SOZ-HFA and SOZ-ISA. In this study, the onset of significant ISA changes was defined as the time at more than + 1 mV or less than − 1 mV. In previous studies^15^, the onset of ISA was defined as the starting time of the ISA change. Therefore, our ISA onset time may be later than the values reported in previous studies. The percentage of SOZ-ISA in this study (88.89%) was concordant with that of a previous report (87%)^4^.

Our proposed PAC brought new insight into the relationship between ISA and HFA. In this study, ISA-HFA PAC reached its maximum in the preictal state, and began changing before SO. In the phase-based analyses, we observed differences between preictal-SOZ and preictal-nSOZ. During high values of ISA-HFA PAC, corresponding to the preictal state, the HFA amplitude in SOZ was tuned to the trough of the ISA oscillations, which was concordant with the findings of previous reports indicating that, during high PAC values, the HFA amplitude is time-locked to the trough of lower frequency oscillations^21,22^. However, during the preictal state, the HFA amplitude of the nSOZ was tuned at the peak of the ISA oscillations. These contrasting trends may reflect neurophysiological differences between SOZ and nSOZ in the preictal state.

In this study, ISA-HFA PAC increased before the increase in HFA occurred, and this profile that PAC precedes HFA increasing is also reported in previous studies related to motor-related physiological PAC^25,26^. A hold-and-release model was proposed, which indicated that physiological coupling restricted the HFA and attenuation of the coupling releases the HFA. We showed that the ISA-HFA PAC before SO was positively correlated with later HFA; therefore, whereas it is unclear whether attenuation of ISA-HFA PAC released seizure-related HFA or not, we inferred that ISA-HFA PAC may have an essential role in inducing an HFA burst during seizures. The mechanisms involved in the transition from interictal to ictal states was reported to be associated with paroxysmal depolarization shift^33^ or spreading depolarization^34^. The time-lag between ISA-HFA PAC appearance and HFA and ISA appearance may represent the transition period from the interictal to ictal state, and ISA-HFA PAC may be a new clue for elucidation of the interictal-ictal transition mechanism.

Various features that are calculated by using encephalography have been proposed for seizure prediction^35^, and machine learning methods have been used to detect preictal states^36,37^. We observed a statistical significance between the interictal and preictal stages in SOZ-PAC, nSOZ-PAC, and SOZ-ISA (Fig. 3). The AUC for the preictal state versus the interictal state was large in this order (Fig. 6). The AUC of SOZ-PAC showed the highest values. Therefore, SOZ-PAC could be a potential, novel biomarker for discriminating between the interictal and preictal states. By using ISA-HFA PAC, achieving accurate and high-performing markers without complex algorithms and long training times is possible. Furthermore, we inferred that an accurate distinction between preictal states and interictal states may allow seizure prediction. Accurate seizure prediction has advantages for responsive neurostimulation with implantable devices^38,39^.

Several features and algorithms have been used to detect seizures^35^, including the PAC of the β band^23^. ISA and HFA occur simultaneously during seizures, as previously reported^4,15,32^. Our study added to the results by demonstrating that SOZ-ISA has a higher performance in differentiating between the ictal and the interictal or preictal states than does SOZ-HFA. ISA and HFA are useful for detecting SOZ^4,15,32^. We also showed that in the ictal state, ISA performed better than HFA for SOZ differentiation.

Contrary to one expectation in our study, the SOZ-HFA amplitude was significantly lower than the nSOZ-HFA amplitude in the interictal states. The interictal-HFO has been reported as less correlated with the ictal onset zone^32^. Therefore, interictal-HFA may be suppressed more in the SOZ than in the nSOZ. This surprising result further contributed to the discrimination between the SOZ and the nSOZ in the interictal state.

This study has some limitations. First, we obtained results by using iEEG, and whether the same results can be obtained using scalp EEG, which is the most commonly used method, is unclear. Stable measurement of extreme frequencies such as ISA and HFA is feasible by iEEG, but not by scalp EEG or magnetoencephalography. This may prevent wider applications in a clinical context. Second, we evaluated only focal-onset seizures. Therefore, whether the same results would be obtained with generalized-onset seizures such as absence or myoclonic seizures remains unclear. Third, our simulation data indicated that ISA-HFA PAC may produce artificially high values because of the methodology itself. The iEEG data without seizures showed no increase in the ISA-HFA SIm; therefore, we considered that the increase in ISA-HFA PAC value was not artificial. In addition, whereas we used a 1-s time window for calculation of SI, the time window that covers at least one full cycle of the lower frequency signals is generally used. We infer that a longer time window may be suitable for neurophysiological elucidation of ISA-HFA PAC phenomenon. To validate whether ISA-HFA PAC was induced by a neuropathophysiological mechanism or by the PAC methodology itself needs further investigation. Fourth, we must consider that external high-frequency noise induced the ISA-HFA PAC increase. Fifth, our sample size was small. Therefore, we evaluated all seizures without adjusting for the number of seizures in each patient. Finally, most seizures were observed during sleep. The consciousness state such as wakefulness or sleep may influence ISA-HFA PAC. However, we could not evaluate this factor because of the small sample size. Further large-scale investigations are needed to prove the usefulness of the presented new method.

In conclusion, ISA-HFA PAC related to focal-onset seizures achieved significantly higher values in the preictal state than in the interictal state and ictal state. ISA-HFA PAC in the SOZ began increasing approximately 87 s (median) before SO, whereas ISA and HFA increased after SO. The receiver-operating characteristic (ROC) curve analysis showed that the ISA-HFA PAC of the SOZ well discriminated between the preictal and interictal states.

## Methods

### Study setting and participants

This retrospective study was performed at Osaka University Hospital in Suita, Osaka, Japan, from July 2018 to July 2019. The study design was approved by the Ethics Committee of Osaka University Hospital (approval no. 19193). Informed consent was obtained from participants using the opt-out method on our center’s website. We confirmed that all methods were performed in accordance with the relevant guidelines and regulations. We included seven patients with drug-resistant focal epilepsy who underwent intracranial electrode placement as part of a presurgical invasive EEG study (Table 1). The patient group in this study was the same group used in our other published study^22^.

### Intracranial electrodes

To acquire iEEG data, we used a combination of subdural grids (10 contacts, 20 contacts, or 30 contacts), strips (four or six contacts), and depth electrodes (six contacts) (Unique Medical Co. Ltd., Komae, Tokyo, Japan), which were placed using conventional craniotomy. The diameter of each contact was 3 mm or 5 mm, and the intercontact distances were 5 mm, 7 mm, or 10 mm for the grid and strip electrodes. The diameter was 1.5 mm and the intercontact distance was 5 mm for the depth electrodes. The location and laterality where the intracranial electrodes were placed were determined by presurgical examinations such as scalp EEG, magnetic resonance imaging, and fluorodeoxyglucose-positron emission tomography. The total number of implanted contacts are shown in Table 1.

### Data acquisition and preprocessing

We acquired iEEG signals using a 128-channel digital EEG system (EEG 2000; Nihon Kohden Corporation, Shinjuku, Tokyo, Japan) at a sampling rate of 1 kHz and a time constant of 10 s. BESA Research 6.0 software (BESA GmbH, Grafelfing, Germany) preprocessed the raw signals using a 60-Hz notch filter with 2 Hz width to eliminate the alternating current line artifact and a zero-phase low-pass filter at 333 Hz with 24 dB/oct slope to prevent aliasing. Next, the BESA software exported the data as a text file. This text file containing iEEG signals was imported into MATLAB R2020a (MathWorks, Natick, MA, USA) and iEEG signals were digitally re-referenced to the common average of all implanted contacts in each patient. The common average was calculated from the mean of all contacts, and the common average was subtracted from each value acquired from each contact.

We saved iEEG data every 60 min; therefore, one text file contained one 60-min signal. We applied a bandpass filter to the entire 60-min data to prevent edge-effect artifacts.

### Seizure onset and contacts

**Figure 7:**
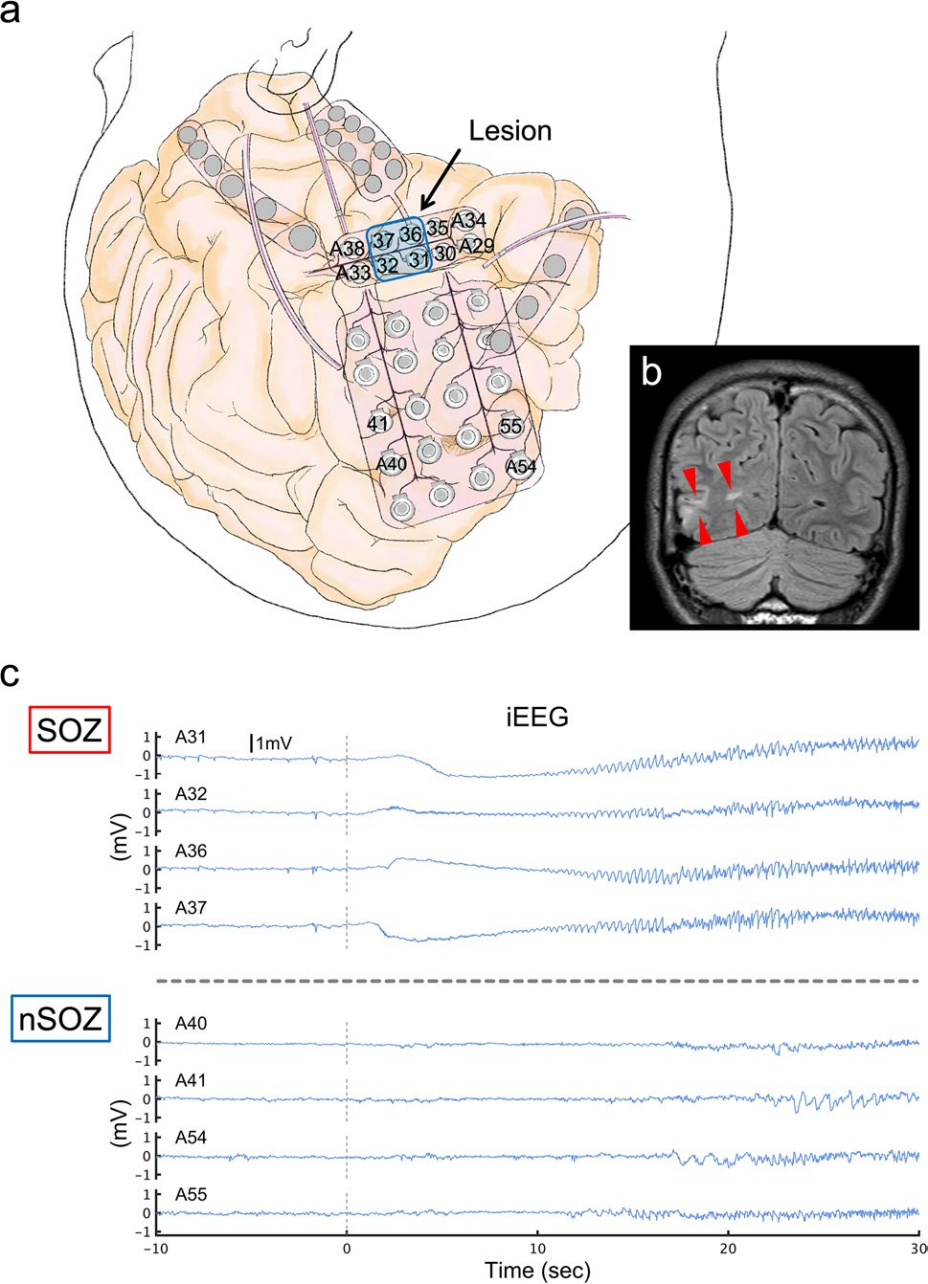
Contacts related to SOZ and nSOZ (P5-S3). (**a**) Illustration of implanted electrodes and the brain are displayed. The numbers correspond to the contacts’ number. This illustration was drawn by Hiroaki Hashimoto using Procreate (Savage Interactive Pty Ltd, Hobart, Australia, https://procreate.art/ipad). (**b**) The high-intensity lesion in the right occipital lobe is shown on the fluid-attenuated inversion recovery MRI (red wedge arrows). This lesion is indicated by the arrow in (**a**). (**c**) The SOZ contacts, which include A31, A32, A36, and A37, placed on the lesion initially show infraslow activity immediately after SO (0 s), followed by low-voltage fast waves. At that time, nSOZ contacts of A40, A41, A54, and A55 show no epileptic changes. The number of SOZ and nSOZ contacts are the same.

The SO was determined by conventional visual inspection of the iEEG signals^40^. The contacts that showed initial epileptic changes immediately after the SO were determined as the SOZ contacts, and the other contacts were determined as the nSOZ contacts. Therefore, most implanted contacts were sorted as nSOZ contacts. For the statistical adjustment, we arranged for the number of SOZ and nSOZ contacts to be the same. To avoid selection bias, we randomly chose the nSOZ contacts (Table 1 and Fig. 7).

The time before the SO was the preictal state and the time after the SO was the ictal state. We defined the interictal state as the seizure-free period, except for 60 min before and 60 min after the SO. We randomly extracted the timepoints from the interictal iEEG data so that the count of timepoints was ten times that of the SO.

### Infraslow activity and high-frequency activity

To extract ISA and HFA, we used 0.016–1 Hz and 80–250 Hz bandpass filters, respectively. A bandpass device with a two-way, least-square, FIR filter (pop_eegfiltnew.m from the EEGLAB toolbox, https://sccn.ucsd.edu/eeglab/index.php) was applied to the iEEG signals. The filter order was automatically set using the function pop_eegfiltnew.m from the EEGLAB toolbox. The oscillations of the 0.016–1 Hz signals were representative of ISA (Fig. 1b). We calculated the amplitude of HFA, which was an envelope of 80–250 Hz signals, in combination with the Hilbert transformation.

### PAC analysis

The SI^18^ was used to measure the strength of PAC between the HFA amplitude and the ISA phase. Hilbert transformation was performed on the bandpass-filtered signals to obtain complex-valued analytic signals [Z(t)]. The amplitude [A(t)] and phase [φ(t)] were calculated from the complex-valued signals using Eq. (1):

1 $$Z\left(t\right)={A} \left(t\right) \cdot \mathit{exp}\left(i \phi \left(t\right)\right)$$

The ISA phase was calculated using the angle of the Hilbert transformation in the 0.016–1 Hz bandpass-filtered signal. The HFA power amplitude was calculated using the squared magnitude of the envelope of the Hilbert transformation in the 80–250 Hz bandpass-filtered signal. The HFA power amplitude time series was normalized, and the phase of this amplitude was then computed using the Hilbert transformation. The SI was calculated using Eq. (2):

2 $$SI= \frac{1}{n} \times \sum_{t=1}^{n}{e}^{i}[ {\phi }_{ISA}\left(t\right) - {\phi }_{HFA}(t)]$$

in which *n* is the number of data points. We used the 1-s time window to calculate the SI in combination with the 1 − k sampling rate. Therefore, *n* in this study was 1000. The SI is a complex number; therefore, we used the SIm in our calculations. The SIm varies between 0 and 1 with 0 indicating completely desynchronized phases and 1 indicating perfectly synchronized phases.

The SIp was calculated by using the arctan (image [SI]/real [SI]). SIp varies between − 180° and + 180°. SIm and SIp values were calculated repeatedly by shifting the 1-s time window every 33 ms, and their time-series data were obtained.

### Bootstrapped technique and family-wise error-corrected threshold

For the statistical assessment of SIm, we shifted the phase-time series of the HFA amplitude and calculated the bootstrapped SIm (SImb) by using the lower frequency phase. We repeated this procedure 1000 times to create the distribution of SImb^18^, which was the surrogate data. The maximum values of the distribution of SImb were stored at each surrogate data, and the distribution of maximum values was created. The values at 95% of the distribution of the maximum were defined as family-wise error (FWE)-corrected threshold, and we applied the FWE-corrected threshold to the observed SIm for the solution to multiple comparisons^41^. SIm values over the FWE-corrected threshold were statistically significant.

### Topographies of HFA, ISA, and SIm

The 0.016–1 Hz bandpass-filtered signals for ISA, 80–250 Hz bandpass-filtered signals for HFA, and SIm signals for PAC were analyzed from 5 min before to 5 min after the SO. The power of the HFA was the square of the HFA amplitude (i.e., envelope) and the series was normalized by HFA power in the initial 1 min (from − 5 to − 4 min before the SO). The HFA-normalized power is presented as a topography (Fig. 2a). We defined the significant seizure-related ISA as oscillations of the 0.016–1 Hz bandpass-filtered signals less than − 1 mV or more than 1 mV; the significant ISAs were displayed in grayscale topography (Fig. 2b). Statistically significant SIm values, acquired by a combination of the bootstrapped technique and FWE-corrected threshold, were indicated in the topography (Fig. 2c).

### Simulation

To assess the SIm between ISA and HFA, we used simulation data. We made 60-min simulation sinusoids for which the frequency was 4 Hz and the amplitude was 5 μV. At 50 min, we inserted three minutes of sinusoids of 200 Hz and 50 μV, which represented the HFA, and 0.016 Hz and 2000 μV sinusoids, which represented ISA. We also added white noise. We calculated ISA-HFA SIm by using the simulation signals.

### Profiles of HFA, ISA, and PAC

For the within-HFA comparisons (Fig. 3a), we used the amplitude of HFA signals, which were an envelope of 80–250 Hz bandpass-filtered signals. For the within-ISA comparisons (Fig. 3b), the absolute values of oscillation of 0.016–1 Hz bandpass-filtered signals were used because the ISAs have slow-negative or slow-positive activities. For the within-PAC comparisons (Fig. 3c), we used the SIm between the ISA phase and HFA amplitude. We averaged the values, using a 30-s time window. The time interval of 30 s before the SO corresponded to the preictal state, the time interval of 30 s after the SO corresponded to the ictal state, and the time interval of 30 s after the timepoint of the interictal period corresponded to the interictal state.

### Time at significant HFA, ISA, and PAC changes

To investigate the time when significant seizure-related changes occurred, we evaluated seizure-related signals from 5 min before to 2 min after the SO. To detect significant changes in HFA, we used a permutation test^42^ to compare the initial 10-s data and the next 10-s data of the HFA-normalized amplitude, which was normalized by the initial 10-s data. Each permutation test produced a set of differences between the initial 10-s data and the next sequential 10-s data. The maximum value of the differences from each permutation test was stored, and the values at 95% of the distribution of these maximum values were considered the FWE-corrected threshold. The values above the FWE-corrected threshold were statistically significant^41^ and the timepoints when the HFA amplitude first crossed over the FWE-corrected threshold were defined as a significant change in the HFA (Supplementary Fig. S7a). However, if the HFA amplitude temporally crossed over the FWE-corrected threshold because of a single-spiking activity, we excluded it from the analysis.

For ISA, the time when iEEG signals first crossed under − 1 mV or over + 1 mV^1,28^ was defined as a significant change in the ISA (Fig. 1a and Supplementary Fig. S7b).

For PAC, we used significant SIm values, which were obtained in combination with the bootstrapped technique and FWE-corrected threshold. If the significant SIm formed a cluster (i.e., significant SIm occurred several times in 30-s time duration), we considered the first timepoint as the significant timepoint of PAC changes (Supplementary Fig. S7c).

### Phase-conditioned analysis

We calculated the mean vector and performed the Rayleigh test to evaluate the nonuniformity of SIp using the CircStat toolbox^43^. To identify the ISA phase to which the HFA amplitude was coupled, we next calculated the average oscillations of ISA and the normalized amplitude of HFA within each ISA phase bin of 30°: − 180° to − 150°, − 150° to − 120°, …, and 150° to 180°.

### Correlation analysis

By using all implanted contacts, we calculated Spearman correlation coefficients between ISA-HFA SIm and the normalized amplitude of HFA (Supplementary Fig. S6).

### Classification

We implemented a classification to distinguish between the preictal and interictal states, the ictal and interictal states, or the ictal and preictal states using HFA amplitude, ISA, and ISA-HFA SIm of the SOZ contacts or nSOZ contacts. We also differentiated between SOZ and nSOZ of the interictal, preictal, and ictal states by using the HFA, ISA, and SIm values. We set the threshold and a binary classification was performed. We obtained a confusion matrix, and sensitivities and specificities were calculated. The ROC curve and its AUC were used to compare the performance of different classifiers.

### Statistical analysis

For the nonparametric and unpaired comparisons between the two groups, we used Wilcoxon rank-sum test (Figs. 3 and 4). For the nonparametric and paired comparisons between the two groups, we used Wilcoxon signed-rank test (Fig. 3 and Supplementary Fig. S5). For the comparison of three groups, we used a one-way analysis of variance (Supplementary Fig. S4. The AUC was also compared, using Wilcoxon signed-rank test. The results were corrected using the Bonferroni correction for multiple comparisons.

## Supplementary Information

Supplementary Information.

## Data Availability

All data that were generated or analyzed in this study are available from the corresponding authors on reasonable request and after additional ethics approval regarding data provision to individual institutions.
