# Supplementary material for: Phase-amplitude coupling between infraslow and high-frequency activities well discriminates between the preictal and interictal states
Source: Sci Rep. 2021 Aug 31;11:17405. doi: 10.1038/s41598-021-96479-1 (PMC8408139; doi:10.1038/s41598-021-96479-1)
Supplement: Supplementary file 1 — Supplementary Information. [file 41598_2021_96479_MOESM1_ESM.pdf]

Supplementary Files

Phase-amplitude coupling between infraslow and high-frequency activities well discriminates between the preictal and interictal states

Hiroaki Hashimoto. MD, PhD [1,2]*; Hui Ming Khoo. MD, PhD [3]; Takufumi Yanagisawa. MD, PhD [3]; Naoki Tani. MD, PhD [3]; Satoru Oshino. MD, PhD [3]; Haruhiko Kishima. MD, PhD [3]; Masayuki Hirata. MD, PhD[1,3]

[1] Department of Neurological Diagnosis and Restoration, Graduate School of Medicine, Osaka University, Suita, Osaka 565-0871, Japan

[2] Department of Neurosurgery, Otemae Hospital, Osaka, Osaka 540-0008, Japan

[3] Department of Neurosurgery, Graduate School of Medicine, Osaka University, Suita, Osaka 565-0871, Japan

Supplementary Figure S1
Supplementary Figure S2
Supplementary Figure S3
Supplementary Figure S4
Supplementary Figure S5
Supplementary Figure S6
Supplementary Figure S7

For all supplementary figures: FWE, family-wise error; HFA, high-frequency activity; iEEG, intracranial electroencephalogram; ISA, infraslow activity; SIm, magnitude of the synchronization index; nSOZ, non-seizure onset zone; PAC, phase-amplitude coupling; SI, synchronization index; SO, seizure onset; SOC, seizure-onset zone contact; SOZ, seizure-onset zone

Supplementary Figure S1. Simulation and noise-contamination results.
These figures have been obtained by using the same methodology described in Figure 1. From the top to the bottom graphs: the raw signals are in black; 0.016–1 Hz band-pass filtered signal, in green; 80–250 Hz band-pass filtered signal, in red; and the ISA-HFA SIm as the PAC, in blue. (a) The evaluation of the simulation data. The data were created by using 4 Hz and 5 μV amplitude sinusoids, which include white noise. At 0 minute, 200 Hz and 50 μV sinusoids, representing the HFA, and 0.016 Hz and 2000 μV sinusoids, representing the ISA, were inserted for 3 minutes. The timepoint of 0 minute represents the SO. High SIm values occurred around 0 minute. Moreover, from -20 minutes to -5 minutes, high SIm values occurred intermittently (red dashed rectangle). This result suggests that the calculation of SIm may have resulted in artificially high values. (b) The results of data contaminated by external high-frequency noise (red arrow) are shown. High ISA-HFA SIm values can be measured in relation to the external high-frequency noise.

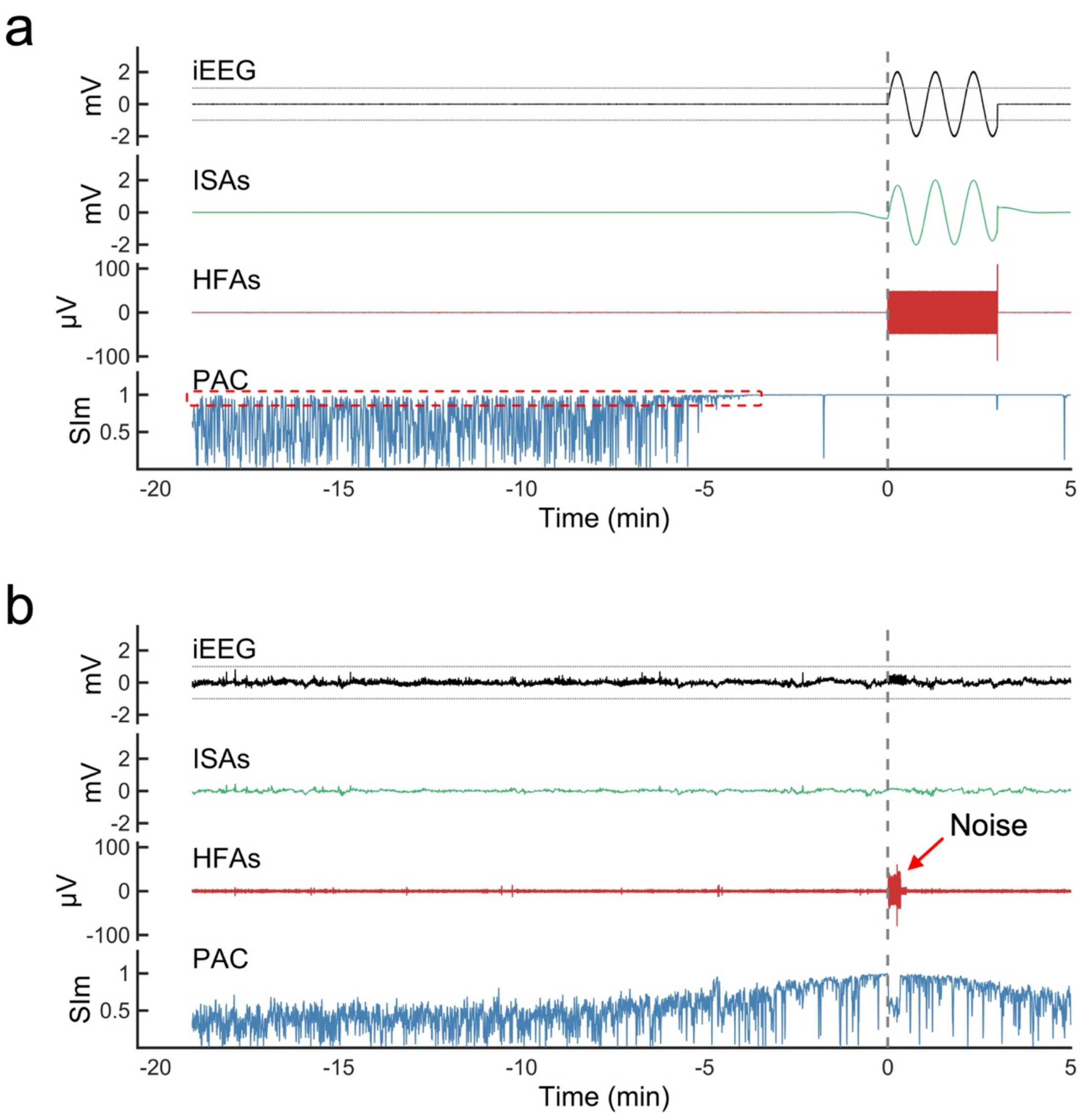

Supplementary Figure S2. Comparison between the seizure file and the nonseizure file. The top to bottom graphs display the results for the HFA-normalized power, the SIm between ISA phase and HFA amplitude, and the SIm between the θ band (4-8 Hz) phase and the HFA amplitude. In our previous study [1], we showed θ-HFA PAC was related to seizure evolution. Therefore, the θ-HFA SIm is the reference. The time-series were obtained with 60 minutes of iEEG data. The results of one SOZ contact (SOC) is indicated in red, and the results of the average of all implanted contacts are in blue. (a) A file that included one seizure was used. This seizure is the same seizure described in Figure 1, Figure 2, and Supplementary Figure S1. Red dashed lines indicate the SO. After SO, the HFA-normalized power and θ-HFA SIm both achieve high values. However, ISA-HFA SIm starts to increase before the SO, and achieves its maximum value at SO. (b) A nonseizure file was used. As expected, no HFA increase occurs. ISA-HFA SIm also does not increase. We were able to confirm that, if iEEG data do not include seizure, the ISA-HFA SIm values cannot be high. The results of the SOC and the average have the same tendency.

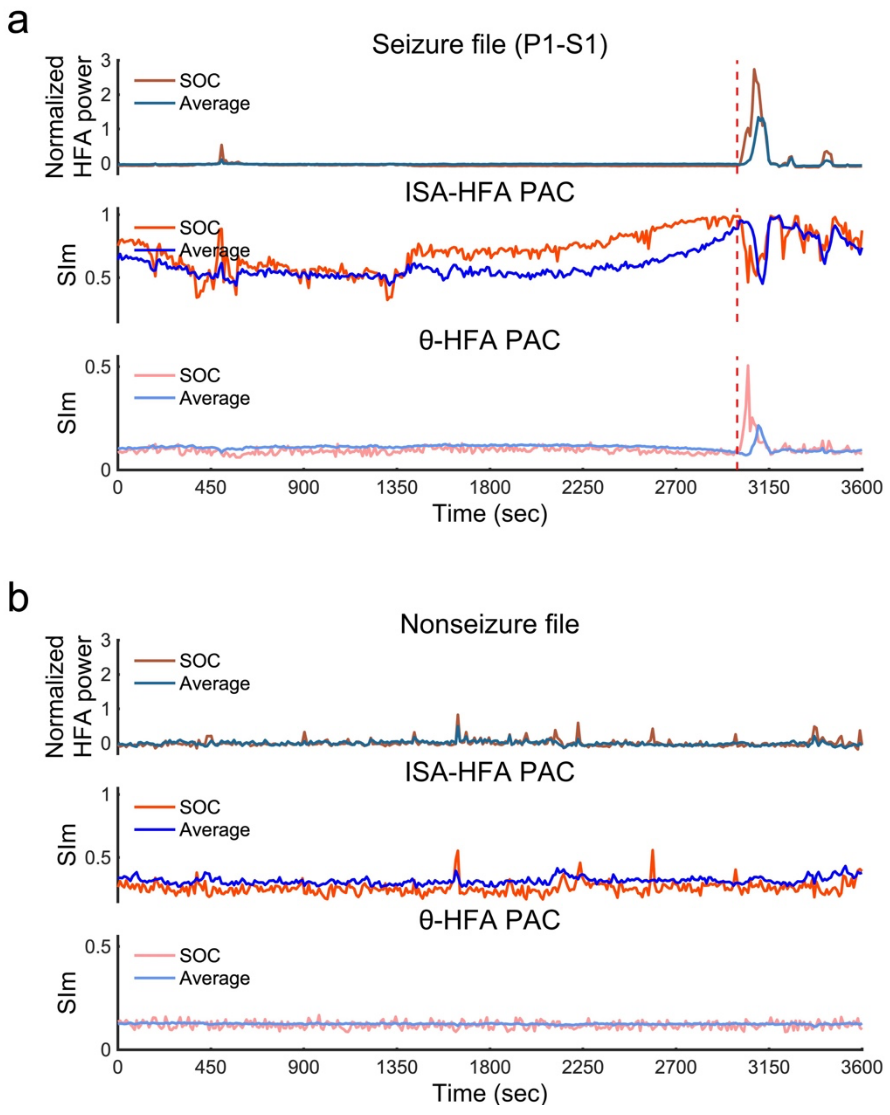

Supplementary Figure S3. Dynamic changes of ISA-HFA SIm calculated using the 1-second time window, 30-second time window, and 63-second time window.

The dynamic changes in the ISA-HFA SIm, calculated by using the 1-second time window (blue), 30-second time window (red), and 63-second time window (green), are plotted (left panels). All 15 seizures are displayed. Red dashed lines indicate the SO. The SIm values were obtained from the averaged values of the total implanted contacts. ISA-HFA SIm values of the 1-second time window are higher than those of the 30-second and 63-second time windows. The same tendency of the SIm beginning to increase before SO occurs in all time window groups. ISA-HFA SIm values were normalized using averages and standard deviations calculated from the 60-minute iEEG data that contained each seizure (right panels). Time-series of all groups almost overlapped.

Supplementary Figure S3-1.

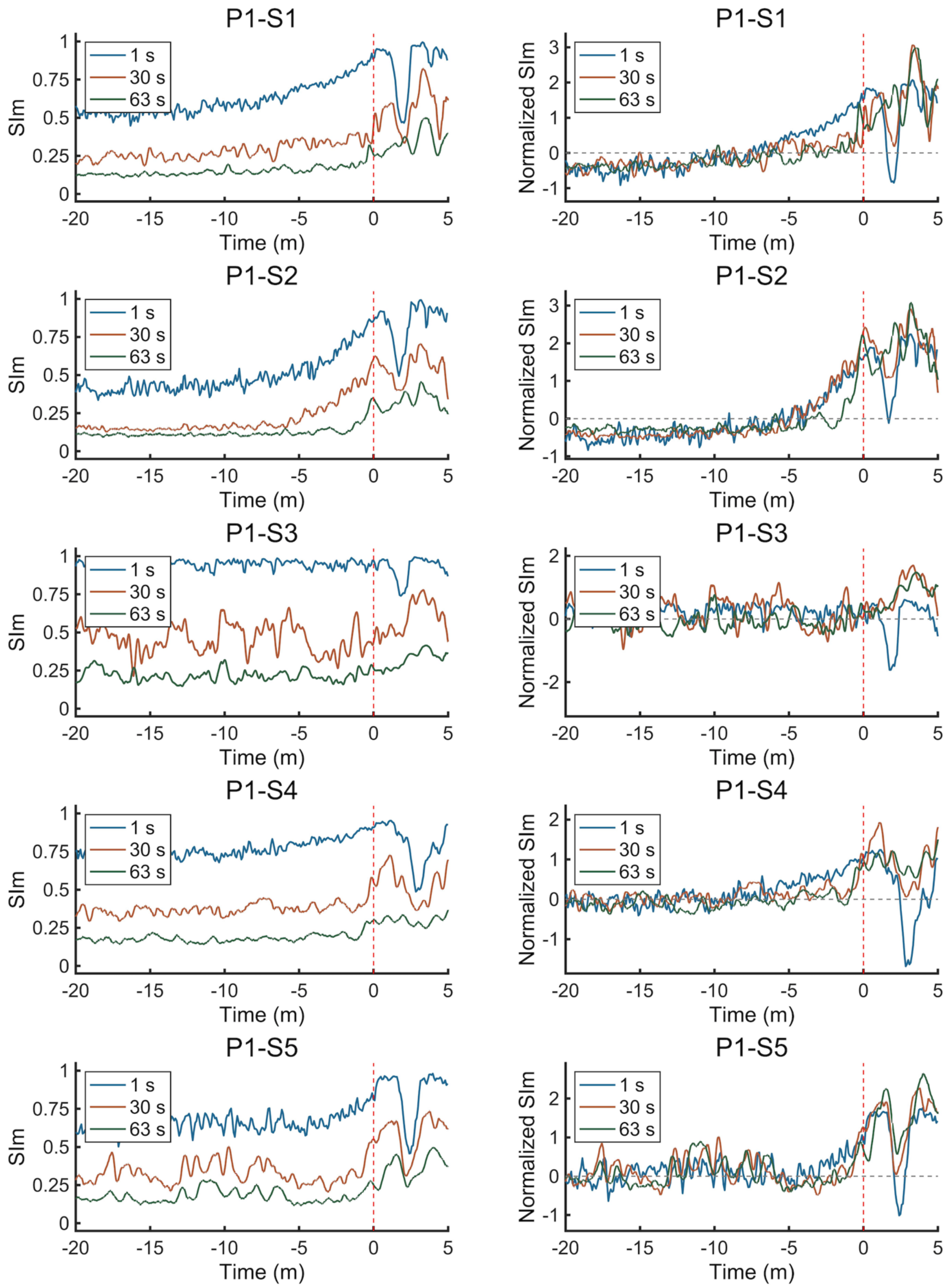

Supplementary Figure S3-2.

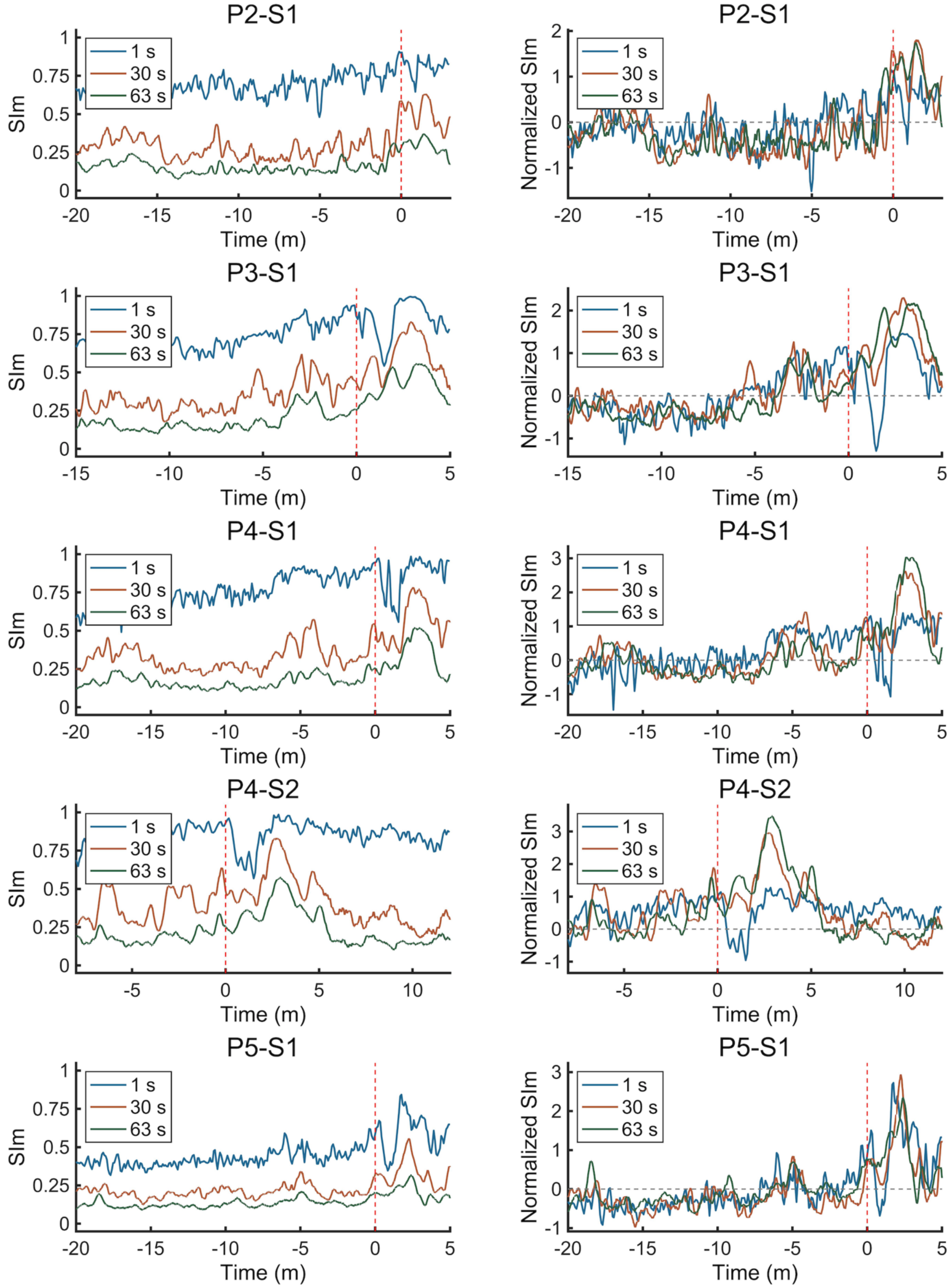

Supplementary Figure S3-3.

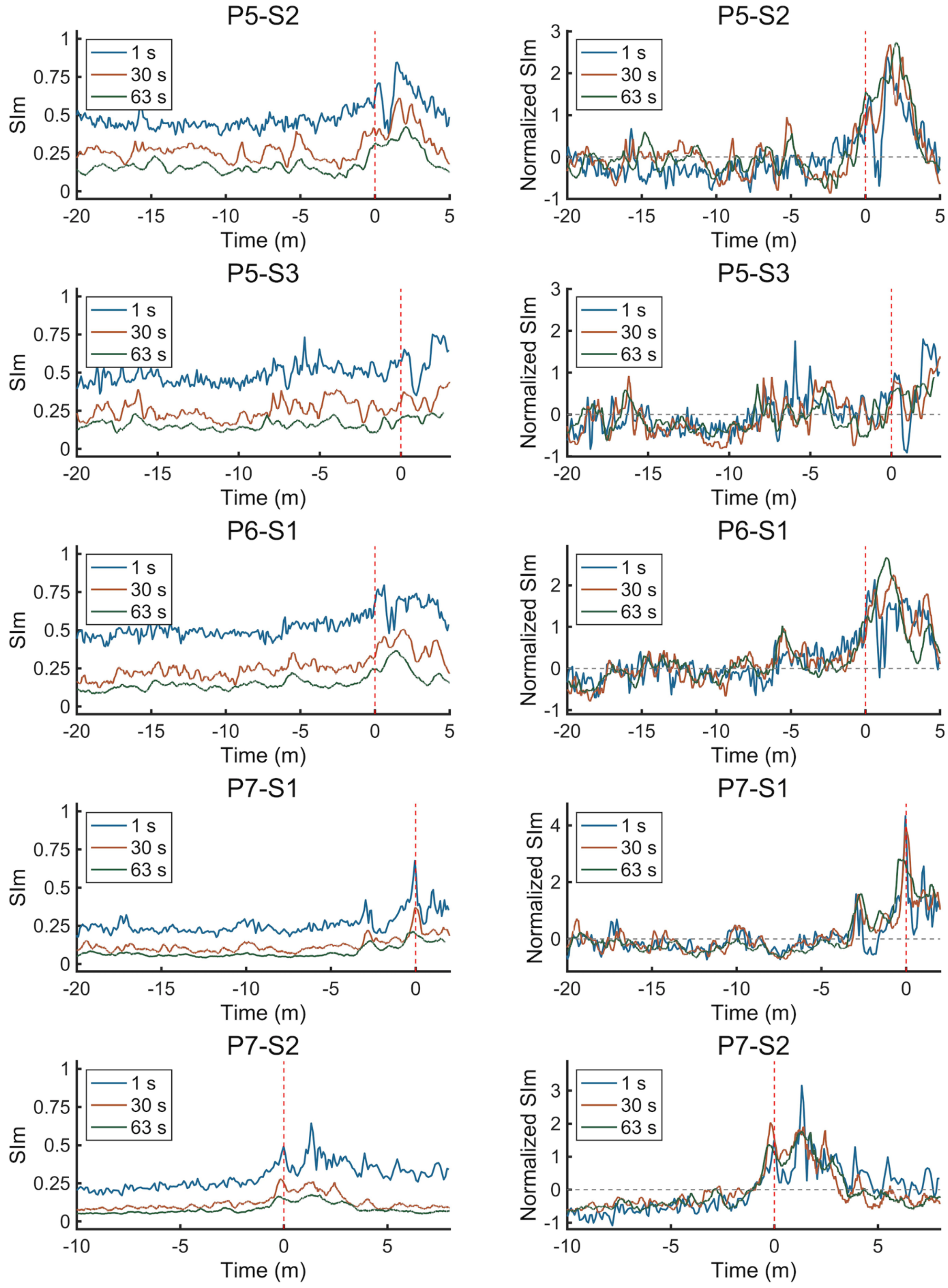

Supplementary Figure S4. Comparison between the 1-second time window, 30-second time window, and 63-second time window at seizure onset.

The ISA-HFA SIm at seizure onset were calculated using the 1-second, 30-second, and 63-second time window. Raw SIm values were compared and significant differences were observed among three groups (one-way analysis of variance, $p = 5.04 \times 10^{-14}$). The longer the time window used, the lower SIm values became (a). However, normalized SIm, calculated by averages and standard deviations of the 60-minute iEEG data that contained each seizure, showed no statistically significant differences (one-way ANOVA, $p = 0.82$).

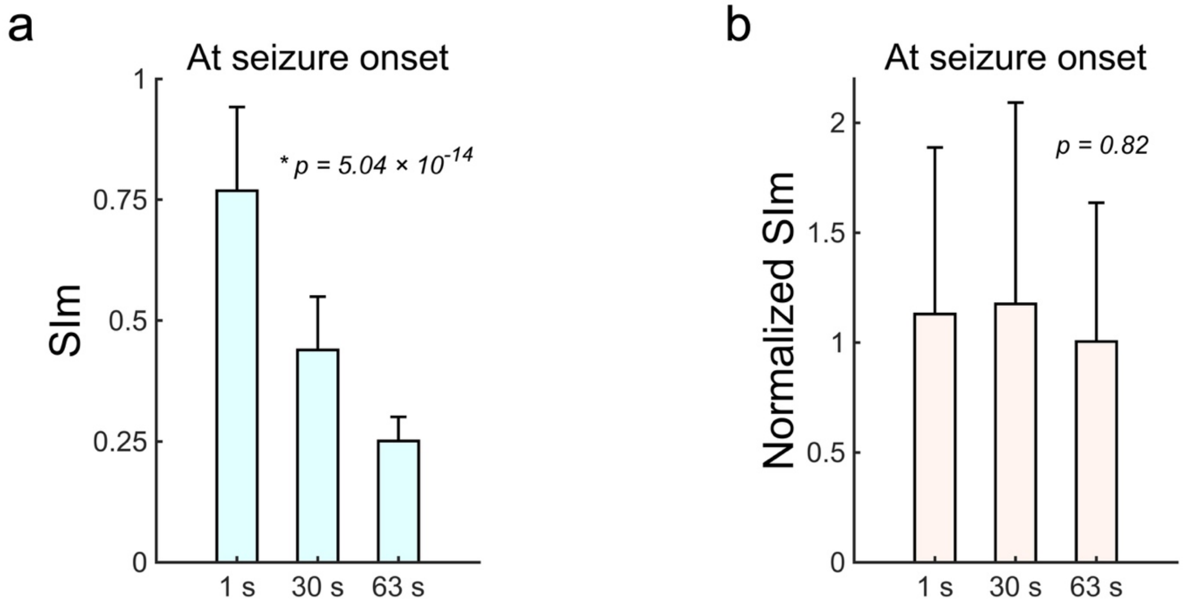

Supplementary Figure S5. The percentages at which significant changes occur.

We evaluated the percentages at which significant changes occur. For HFA and PAC (i.e., ISA-HFA SIm), nearly all SOZ and nSOZ contacts show significant changes. For ISA, 89% of the SOZ contacts and 67% of the nSOZ contacts show significant changes. No significant differences exist between the groups.

Wilcoxon signed-rank test across patients, nine multiple comparisons, corrected by Bonferroni method.

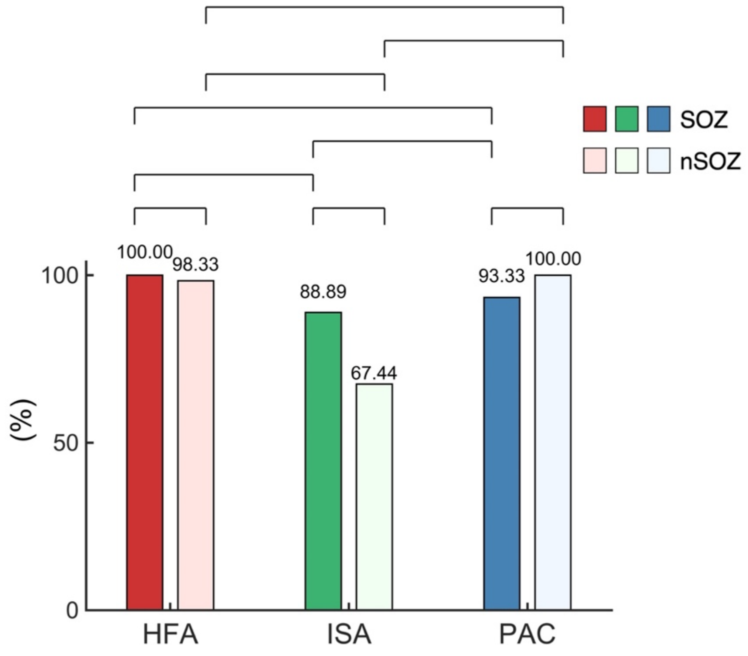

Supplementary Figure S6. Correlation between HFA and PAC.

Correlation coefficients (*r*) and their corresponding significant p values (corrected $p < 0.01$, 40×40 multiple comparisons corrected by Bonferroni method) are shown in panels a and b for all combinations of sequential HFA-normalized amplitude and sequential PAC (ISA-HFA SIm), from -5 minutes to +2 minutes around the SO (0 minutes). We observed a significant positive correlation in the range after SO (for HFA) and before SO (for PAC). Moreover, we observed a negative correlation along the diagonal line from approximately 0 min to 1.5 min (red dashed square in panel a). These figures were created by MATLAB R2020a (MathWorks, Natick, MA, USA, https://www.mathworks.com/products/matlab.html).

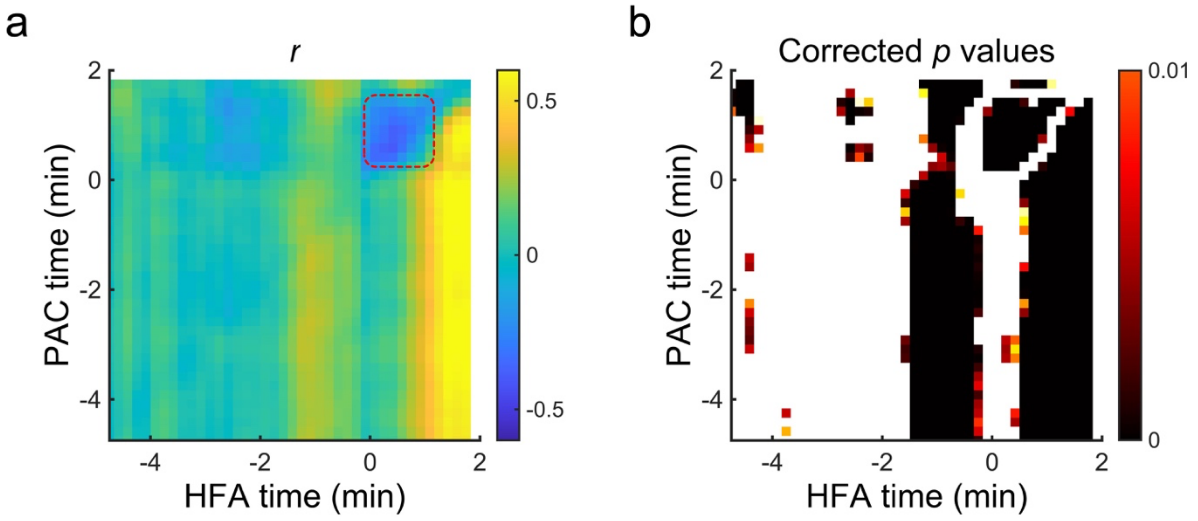

Supplementary Figure S7. Period of significant change in seizure 1 in Patient 1.

The seizure-related data from 5 minutes before to 2 minutes after SO (0 min) of seizure 1 (S1) in Patient 1 (P1) are shown. (a) The HFA-normalized amplitude (red line) and the FWE-corrected threshold (dashed blue line) are indicated. At 39.40 seconds, the HFA-normalized amplitude crossed over the FWE-corrected threshold for the first time, thereby making this timepoint significant. (b) The iEEG signals (green lines) range from -1 mV to +1 mV (gray mesh) are indicated. The timepoint of 7.80 seconds was the significant time of the ISA change when the iEEG signal first crossed under -1 mV. (c) A statistically significant SIm occurred at -171.31 seconds, which was the significant timepoint in the PAC change.

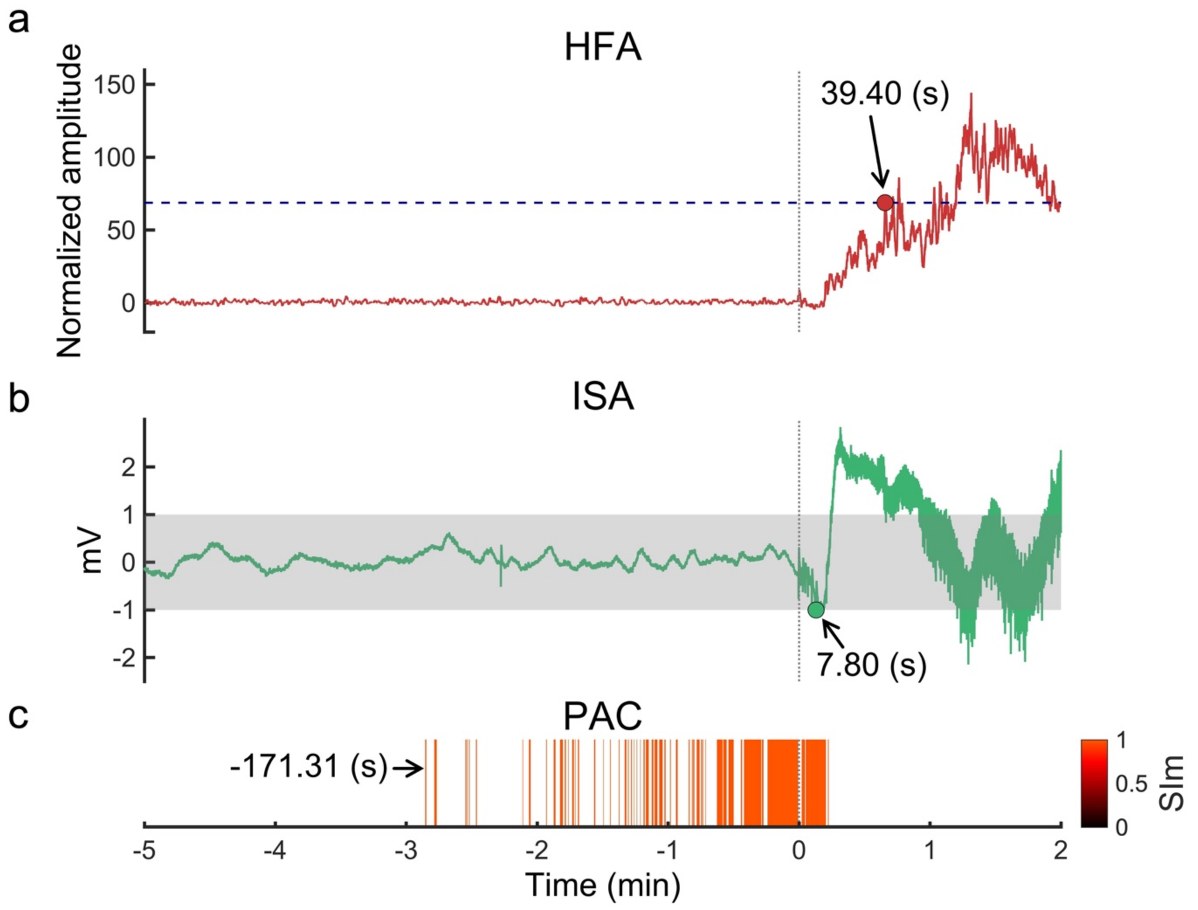

References

1 Hashimoto, H. *et al.* Phase-amplitude coupling of ripple activities during seizure evolution with theta phase. *Clin. Neurophysiol.* **132**, 1243-1253, doi:https://doi.org/10.1016/j.clinph.2021.03.007 (2021).
